# An Uncharacterised lncRNA Coded by the ASAP1 Locus Is Downregulated in Serum of Type 2 Diabetes Mellitus Patients

**DOI:** 10.3390/ijms241713485

**Published:** 2023-08-30

**Authors:** Cristina Barbagallo, Michele Stella, Stefania Di Mauro, Alessandra Scamporrino, Agnese Filippello, Francesca Scionti, Maria Teresa Di Martino, Michele Purrello, Marco Ragusa, Francesco Purrello, Salvatore Piro

**Affiliations:** 1 Section of Biology and Genetics, Department of Biomedical and Biotechnological Sciences, University of Catania, 95123 Catania, Italy; cbarbagallo@unict.it (C.B.); michelestella7@gmail.com (M.S.); purrello@unict.it (M.P.); 2 Department of Clinical and Experimental Medicine, Internal Medicine, Garibaldi-Nesima Hospital, University of Catania, 95122 Catania, Italy; 8stefaniadimauro6@gmail.com (S.D.M.); alessandraska@hotmail.com (A.S.); agnese.filippello@gmail.com (A.F.); fpurrell@unict.it (F.P.); salvatore.piro@unict.it (S.P.); 3 Department of Experimental and Clinical Medicine, Magna Graecia University, 88100 Catanzaro, Italy; fscionti20@gmail.com (F.S.); teresadm@unicz.it (M.T.D.M.)

**Keywords:** ncRNAs, diabetes, diagnosis, T2D, biomarkers, predictive preventive personalised medicine (3PM/PPPM)

## Abstract

Diabetes mellitus (DM) is a complex and multifactorial disease characterised by high blood glucose. Type 2 Diabetes (T2D), the most frequent clinical condition accounting for about 90% of all DM cases worldwide, is a chronic disease with slow development usually affecting middle-aged or elderly individuals. T2D represents a significant problem of public health today because its incidence is constantly growing among both children and adults. It is also estimated that underdiagnosis prevalence would strongly further increase the real incidence of the disease, with about half of T2D patients being undiagnosed. Therefore, it is important to increase diagnosis accuracy. The current interest in RNA molecules (both protein- and non-protein-coding) as potential biomarkers for diagnosis, prognosis, and treatment lies in the ease and low cost of isolation and quantification with basic molecular biology techniques. In the present study, we analysed the transcriptome in serum samples collected from T2D patients and unaffected individuals to identify potential RNA-based biomarkers. Microarray-based profiling and subsequent validation using Real-Time PCR identified an uncharacterised long non-coding RNA (lncRNA) transcribed from the ASAP1 locus as a potential diagnostic biomarker. ROC curve analysis showed that a molecular signature including the lncRNA and the clinicopathological parameters of T2D patients as well as unaffected individuals showed a better diagnostic performance compared with the glycated haemoglobin test (HbA1c). This result suggests that the application of this biomarker in clinical practice would help to improve the diagnosis, and therefore the clinical management, of T2D patients. The proposed biomarker would be useful in the context of predictive, preventive, and personalised medicine (3PM/PPPM).

## 1. Introduction

Diabetes mellitus (DM) is a complex and multifactorial disease characterised by high glucose levels in the blood. Hyperglycaemia may be caused by the loss of insulin secretion by pancreatic β-cells or insulin resistance acquired by body tissues; it is also possible that both processes coexist and contribute to disease onset [1]. Type 2 Diabetes (T2D) represents the most frequent clinical condition, accounting for about 90% of all DM cases worldwide. It is a chronic disease with slow development usually affecting middle-aged or elderly individuals, typically deriving from insulin resistance or defects in insulin secretion. T2D develops slowly, frequently showing no symptoms and being undiagnosed in the early phase; when manifest, symptoms include excessive thirst and hunger (polydipsia and polyphagia), polyuria, and weight loss. Insulin secretion is progressively impaired and often aggravates a pre-existent condition of insulin resistance occurring in the liver, skeletal muscle, and adipose tissue [1,2].

T2D represents a significant problem of public health today because its incidence is constantly growing among both children and adults. This increase is caused by several factors, among which are the extension of life expectancy, the “epidemy of obesity” associated with a sedentary lifestyle and excessive or imbalanced nutrition. It is also estimated that the prevalence of underdiagnosis would strongly further increase the real incidence of the disease, with about half of T2D patients being undiagnosed [2,3]. The scenario is exacerbated by the serious clinical complications deriving from DM, traditionally associated with the macro- and micro-vascular system, such as coronary heart disease, heart failure, stroke, peripheral arterial disease, diabetic kidney disease, retinopathy, and peripheral neuropathy. Clinical management of DM patients has recently been improved, leading to a longer life expectancy associated with these complications; however, this has also revealed the emergence of new pathological states associated with DM, including, among others, cancer, liver disease, cognitive disability, and infections [4]. Predictive preventive personalised medicine (3PM/PPPM) would allow for the improvement of clinical management and, consequently, induce benefits in the healthcare system and overall quality of life in society [5,6,7]. The principles of 3PM would also improve the management of T2D complications, such as diabetic retinopathy [8,9]. For these reasons, it is important to increase diagnosis accuracy by identifying the disease in its early stages. Currently, DM diagnosis is based on hyperglycaemia measured under different conditions: fasting plasma glucose test (FPG) ≥ 126 mg/dL; 2 h oral glucose tolerance test (OGTT) ≥ 200 mg/dL; glycated haemoglobin test (HbA1c) ≥ 6.5%; and random plasma glucose test ≥ 200 mg/dL [10]. Prediabetes is defined as a condition of risk for T2D development, characterised by increased levels of glycaemia and HbA1c compared to normal values, but still lower than T2D diagnosis reference values. Prediabetic patients are highly heterogeneous both in pathophysiology and clinical manifestations [1,2].

In the last decades, research on diagnostic biomarkers has focused particularly on non-coding RNAs (ncRNAs), which have been widely investigated in several biological fluids of patients affected by many different diseases [11,12,13,14]. Reports have also shown the application of ncRNAs as diagnostic biomarkers in DM and DM-associated complications [15,16,17,18,19,20,21,22,23]. The use of circulating nucleic acids as biomarkers agrees with the principles of 3PM [24], because of the application of innovative techniques with a diagnostic or predictive purpose [25]. The importance of ncRNAs in physiology and diseases is widely accepted. These molecules are able to regulate crucial processes within cells but are also actively secreted as mediators of cell-to-cell communication participating in disease onset and progression. Specifically, ncRNAs are a heterogeneous class of transcripts that do not undergo translation that can be classified according to their length into long non-coding RNAs (lncRNAs), from 200 nucleotides up to kilobases, and small ncRNAs, ranging from a few to 200 nucleotides. LncRNAs also include circular RNAs (circRNAs), a particular class of transcripts with extremities covalently bound creating a circular structure. LncRNAs have a wide range of functions, including epigenetic regulation at different levels. Among small ncRNAs, the most studied class is microRNAs (miRNAs), endogenous transcripts with a size of 18–25 nucleotides acting as negative regulators of gene expression at the post-transcriptional level [26]. The current interest in RNA molecules (both coding and non-coding) as potential biomarkers for diagnosis, prognosis, and treatment lies in the ease and low cost of isolation and quantification with basic molecular biology techniques. Quantification is particularly easy and low cost according to the chosen biological fluid: blood is among the best fluids to analyse because of its easy and low-cost collection, and low impact on the patient in terms of invasiveness, pain, and side effects. The present study aimed to analyse the transcriptome in serum samples collected from T2D patients and unaffected individuals to identify potential RNA-based biomarkers useful for T2D diagnosis. Such analysis allowed us to select RNA molecules, including both protein- and non-protein-coding RNA molecules, to be potentially applied in clinical practice.

## 2. Results

### 2.1. Profiling of Serum Samples from T2D Patients and CTRL Individuals

**Figure 1:**
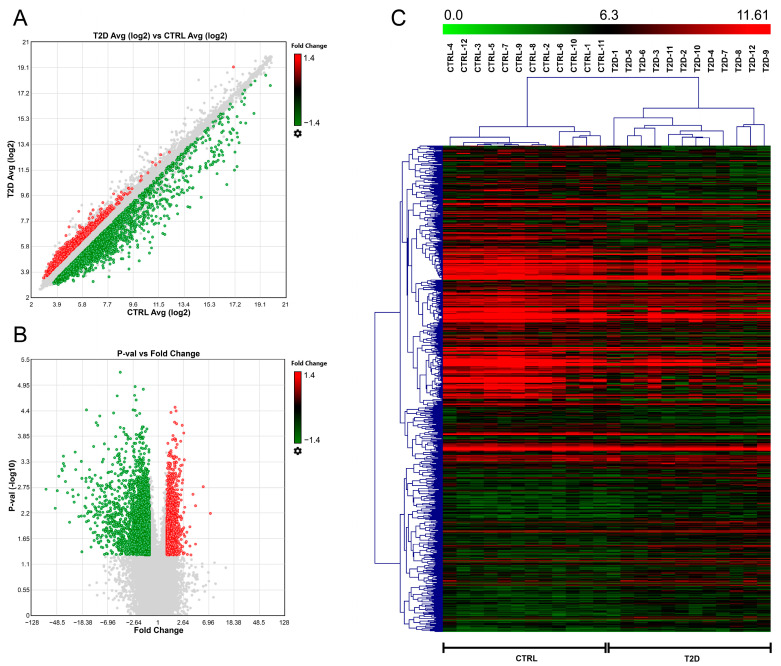
Results of microarray analysis of serum samples from T2D patients and CTRL individuals. (**A**) Scatter plot showing log2-transformed fluorescence intensity values of DE transcripts; (**B**) volcano plot showing *p*-value vs. FC for each DE transcript. Coloured dots represent DE transcripts (*p* < 0.05), where red is upregulation (FC > 1.4) and green is downregulation (FC < −1.4). (**C**) Hierarchical clustering of T2D and CTRL samples according to the expression of DE transcripts reported as fluorescence intensity (*p*-value < 0.005).

The microarray analysis performed with the Clariom D Pico assay compared the transcriptome of 12 T2D patients and 12 CTRL individuals. Results were obtained by applying two statistical approaches (TAC and MeV software, as described in Section 4 “Materials and Methods”). Data were then filtered according to fluorescence intensity, selecting only transcripts showing high-intensity values to ensure signal detection in validation analysis. This profiling analysis showed 5876 DE transcripts, among which 2127 (36.2%) were upregulated and 3749 (63.8%) downregulated (*p*-value < 0.05, FC < −1.4 or FC > 1.4) (Figure 1). Intensity values for all 24 samples are available in supplementary materials.

A subset of DE transcripts was selected for the following validation step. In particular, profiling results were filtered to focus attention on the most promising potential biomarkers for T2D diagnosis. Filtering was performed by selecting transcripts expressed in both DM and CTRL groups and showing the most significant *p*-values, the strongest FCs, and the highest fluorescence intensities. We also removed from the list of potential biomarkers those transcripts with short sequences (<100 nucleotides) or encoded by loci sited in low complexity or repeated regions of the genome. Finally, we selected 17 DE transcripts for validation analysis (Table 1).

### 2.2. Validation of Profiling Results by Real-Time PCR Single Assays

Specific PCR primers were designed for each DE transcript. PCR primers were first tested on a small group of samples to verify if a fluorescence signal was detectable in Real-Time PCR. Four transcripts gave no amplification signal detectable in Real-Time PCR and were consequently removed from the analysis. According to the results, the expression of 13 transcripts was evaluated in an independent cohort of 72 individuals (35 T2D vs. 37 CTRL). Results confirmed the significant downregulation of TC0800011832 according to both endogenous controls (Table 2). All Ct and Tm values are available in the supplementary materials.

**Figure 2:**
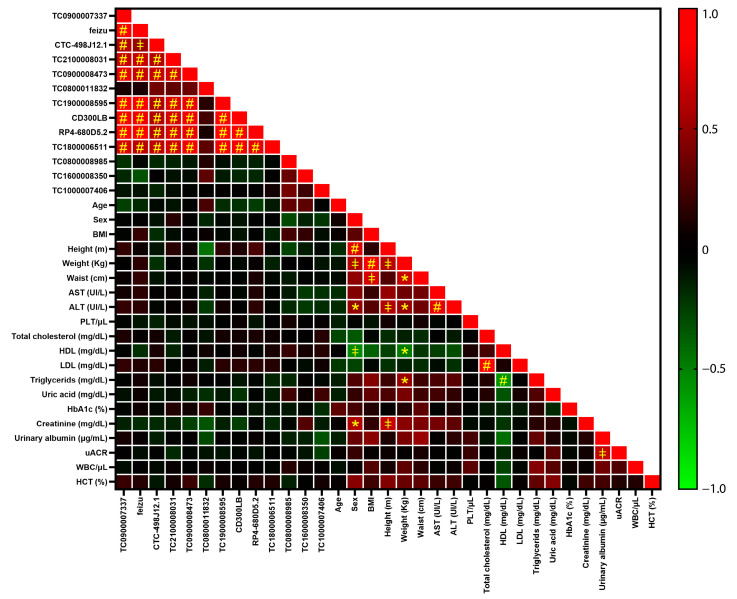
Results of correlation analysis. Correlation coefficients computed between transcript expression data and clinicopathological features of study participants are shown in a colour-coded scale ranging from green (negative correlation) to red (positive correlation. Statistical significance is shown by symbols: *: *p*-value < 0.05; ǂ: *p*-value < 0.005; #: *p*-value < 0.0005.

The association of all analysed transcripts with clinicopathological parameters was evaluated by correlation analysis. No significant correlation was observed between the transcripts (both deregulated and not) and the clinicopathological features of the study participant after correcting *p*-values for multiple comparisons (Figure 2). On the other hand, a highly significant positive correlation of expression was observed among not-deregulated transcripts (Figure 2). Differences among pathological groups were evaluated to assess the presence of confounding factors among clinicopathological parameters that would have affected our analysis. The only significant difference was related to glycosylated haemoglobin, which, as expected, was significantly higher in T2D patients compared to CTRL individuals (*p*-value < 0.0001).

### 2.3. ROC Curves

**Figure 3:**
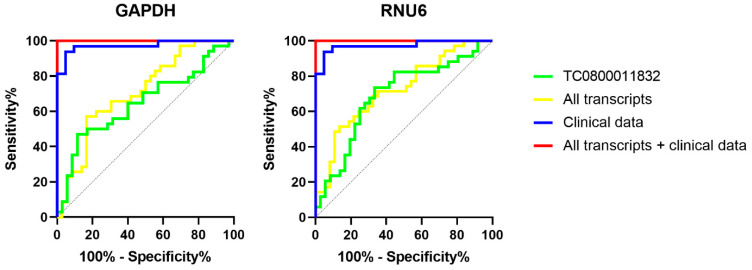
Univariable and multivariable ROC curves computed on expression data and clinicopathological parameters.

Aiming to identify potential diagnostic biomarkers for T2D, the diagnostic accuracy of the validated DE transcript was evaluated by computing ROC curves. A univariable ROC curve was computed for TC0800011832, the only significantly deregulated transcript, showing an AUC inferior to 0.7 with both endogenous controls (GAPDH and RNU6) (Table 3, Figure 3). This value suggests a poor diagnostic performance (about 70% accuracy) for TC0800011832 as a diagnostic biomarker for T2D. In light of this result, we also computed ROC curves for multiple combinations of biomarkers, as it is known that a signature of biomarkers improves diagnostic accuracy compared to single biomarkers [27]. These multivariable ROC curves were computed considering: (i) all analysed transcripts (even if not deregulated); (ii) all available clinicopathological data; (iii) the combination of the DE transcript/all transcripts and clinicopathological data. All results are shown in Table 3 and Figure 3. Clinicopathological data showed excellent diagnostic performance (AUC = 0936), driven especially by HbA1c, which increased the AUC from 0.777 to 0.973 (Supplementary Table S1). A very similar performance was observed for the combination of TC0800011832 and clinicopathological parameters with both endogenous controls. Interestingly, the diagnostic performance further increased when the signature included all analysed transcripts, as well as the ones that were not DE. This result suggests that combining expression data and clinicopathological parameters commonly used in clinical practice would improve the diagnosis of T2D. All ROC curves computed considering several combinations of transcripts and clinicopathological parameters are shown in Supplementary Table S1. The multivariable ROC curves including all transcripts/clinicopathological parameters showed a better performance than the ROC curves built considering only the DE transcript and HbA1c, which are the only biomarkers showing significant differences among the two pathological groups.

## 3. Discussion

Biomarkers measured in blood (or derivative fluids) represent a very advantageous instrument for the clinical management of patients. Biomarkers can be used to diagnose a disease, evaluate the prognosis, or estimate treatment response. The great advantage of blood biomarkers is the ease of collection of the blood sample, with no or very low pain or side effects for the patient. Blood allows the analysis of biomarkers originating from all regions of the human body and is a source of molecules with diverse chemical compositions such as proteins, nucleic acids, and lipids. Among the available biomarkers, RNA molecules are presently regarded as among the most favourable choices. This is due to their ease of isolation from blood samples (serum or plasma) and the feasibility of analysis using simple molecular biology methods, all at a minimal cost. Concerning analysis, the gold standard technique is PCR, with all its variants, which can evaluate expression levels of transcripts with high sensitivity. Accordingly, we investigated the transcriptomic profiles of serum samples from T2D patients and unaffected individuals aiming to identify new potential diagnostic biomarkers for T2D. In this study, we applied a common experimental plan consisting of a discovery phase, performed with a high-throughput microarray technique, followed by a validation step on a larger independent cohort aiming at confirming the results of the profiling analysis.

**Figure 4:**
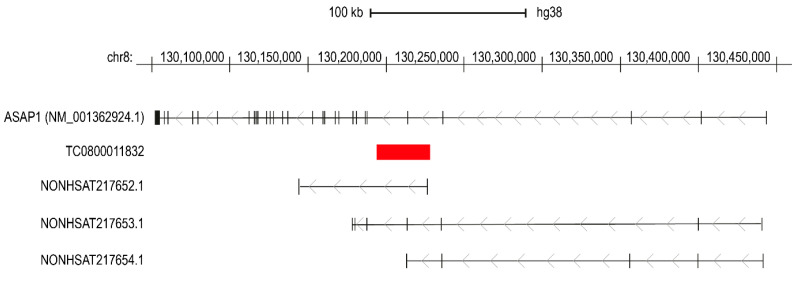
Sequence alignment of TC0800011832 with ASAP1 transcript variant 3 (NM_001362924.1) and ncRNAs from the NONCODE database.

The Clariom D Pico assay evaluates transcriptomic profiles (including several classes of RNA molecules, both coding and non-coding) in clinical samples. This platform represents a valuable approach for identifying RNA-based biomarkers useful as diagnostic and prognostic tools. Moreover, the Pico assay is particularly suitable for the analysis of biological fluids because it requires a very low input mass of total RNA, which is often isolated from fluidic samples in very low quantities. Indeed, some reports analysed biofluids from humans for this purpose [28,29,30,31,32,33]. In this study, we performed transcriptomic analysis through the Clariom D platform on 24 samples obtained from 12 T2D patients and 12 unaffected individuals. According to our double approach for statistical analysis, we identified a set of DE transcripts, which were further tested for their potential application as diagnostic biomarkers for T2D. Validation using Real-Time PCR was performed on a larger cohort of 72 patients (35 T2D vs. 37 CTRL), confirming the significant downregulation of the transcript identified by the TAC ID TC0800011832. Aiming to characterise this transcript, we used the Blat tool within the UCSC Genome Browser (https://genome.ucsc.edu/cgi-bin/hgBlat?command=start, accessed on 28 June 2023) to identify the locus of the human genome from which TC0800011832 is transcribed. The results showed a 100% sequence identity with the locus encoding for ASAP1 (ArfGAP with SH3 domain, ankyrin repeat and PH domain 1) on chromosome 8; specifically, the TC0800011832 coding sequence overlapped the region including part of the fourth intron, the entire fifth exon, and part of the fifth intron of the ASAP1 transcript variant 3 (NM_001362924.1) (Figure 4).

ASAP1 encodes an ADP-ribosylation factor (ARF) GTPase-activating protein involved in cytoskeleton dynamics and remodelling by direct binding with actin [34]. Accordingly, an oncogenic role in different cancer models was proposed for ASAP1, involved in tumour motility, invasiveness, and adhesiveness, finally leading to metastasis [35]. The role of ASAP1 in DM is not clear; however, some evidence showed a potential association with the disease. Increased expression of ASAP1 mRNA was observed in the adipose tissue of obese (ob/ob) and diabetic (db/db) mice compared with wild-type animals, suggesting its involvement in adipogenesis [36]. Another study showed that loss of ASAP1 expression resulted in delayed growth, ossification, and adipocyte development, reducing fat depot [37]. Decreased mRNA expression was also observed in the kidneys of diabetic mice (KK/Ta) [38]. The SNP rs10956514 lying in the coding sequence of ASAP1 is associated with susceptibility to tuberculosis [39] and also moderately associated with T2D (but not with Type 1 Diabetes) risk [40]. There is a link between DM and the cytoskeleton, as a correct organisation of filamentous actin (F-actin) in pancreatic β cells is required for insulin secretion [41]. Moreover, insulin mediates cytoskeleton remodelling in podocytes of obese insulin-resistant rats [42], suggesting that this association is conserved in different cell types. Among DM-induced complications, diabetic cardiomyopathy may derive from an aberrant spatial organisation of F-actin in cardiomyocytes, inducing cell stiffness [43]. It was reported that hyperglycaemia increased the expression of contractile smooth muscle markers (at both mRNA and protein levels) in vascular smooth muscle cells in vitro; however, these were also present in hyperglycaemic mice and in diabetic patients in vivo, and increased glucose levels also induced actin polymerisation. This observation may contribute to the hypercontractile phenotype acquired by the vascular smooth muscle of diabetic patients and animal models [44]. Looking at the discussed evidence, we may speculate an involvement of ASAP1 in DM pathogenetic mechanisms. Further studies will be required to evaluate the effective role of ASAP1 in disease-related processes. By querying the NONCODE database (http://noncode.org/, accessed on 28 June 2023), we identified three uncharacterised ncRNAs (namely NONHSAT217652.1, NONHSAT217653.1, NONHSAT217654.1) partially overlapping the sequence of TC0800011832 (Figure 4). According to NONCODE, NONHSAT217653.1, and NONHSAT217654.1 are expressed as circulating RNAs in the exosomes from the blood of normal individuals; this evidence is congruent with the decreased expression observed in T2D patients compared with unaffected individuals.

In the perspective of applying the transcript TC0800011832 as a diagnostic biomarker for T2D, we evaluated its performance by computing ROC curves. The univariable ROC curve showed poor diagnostic accuracy, thus we evaluated different signatures of multiple biomarkers to assess if the diagnostic performance would have improved. First of all, we considered a signature including all analysed transcripts. Despite 12 out of 13 transcripts showing unchanged expression between T2D patients and CTRL individuals, the signature including all of them together with TC0800011832 showed better diagnostic performance, as shown by the AUC value. This is coherent with the observation that signatures of multiple biomarkers perform better than single biomarkers. Similarly, multivariable ROC curves based on the clinicopathological parameters collected from all study participants showed a higher AUC value when compared with the univariable ROC curve built on HbA1c (Supplementary Table S1). Accordingly, the best combination of biomarkers was the one including all analysed transcripts and clinicopathological data, reaching the maximum AUC value. This result suggests that combining a currently used biomarker such as Hb1Ac may improve the accuracy of T2D diagnosis. Further studies are needed to investigate the efficacy of such signatures in large and multicentric cohorts of patients, strengthening the role of these signatures in 3PM.

## 4. Materials and Methods

### 4.1. Patient Recruitment

This study was conducted according to the Declaration of Helsinki and was approved by the Institutional Review Board of the University of Catania. Written informed consent was obtained from each study participant. A total of 96 individuals were recruited for this study. Serum samples from 24 individuals represented the discovery set, while 72 were analysed as the validation set. The total cohort included 47 patients affected by T2D and 49 unaffected individuals as controls (CTRL). All individuals were aged between 50 and 65, were not affected by systemic pathologies, including cancer, autoimmune and neurodegenerative diseases, and had no history of myocardial infarction or stroke. Inclusion criteria for T2D patients were: (I) diagnosis according to blood glucose levels (random blood glucose ≥ 200 mg/dL and/or fasting blood glucose ≥ 126 mg/dL), and/or HbA1c ≥ 6.5% (48 mmol/mol); (II) absence of diabetes-associated complications including nephropathy, neuropathy, and retinopathy. The clinicopathological parameters of the study participants are shown in Table 4. The statistical power of this study was evaluated by G*Power 3.1 (Universität Düsseldorf, Düsseldorf, Germany): a power of 0.6 and an effect size of 0.8 were estimated for profiling analysis on 24 samples, while a power of 0.7 and an effect size of 0.5 were calculated for validation on 72 samples.

### 4.2. Serum Sample Processing

Peripheral blood was collected from study participants in the morning by venous sampling into separator collection tubes containing a clot activator and gel for serum separation as additives (BD Biosciences, Franklin Lakes, NJ, USA). Serum was separated from blood according to the current procedures for clinical samples [45]. Samples were incubated at room temperature and processed within a maximum of two hours from collection. Serum was obtained by centrifugation at 2000× *g* at 4 °C for 15 min; the resulting supernatant was centrifuged again under the same conditions to ensure the elimination of potential contaminant blood cells. The obtained serum samples were aliquoted in RNase- and DNase-free tubes and stored at −80 °C until analysis. Serum samples were analysed with a Multiscan Ascent microplate reader spectrometer (Thermo Fisher Scientific, Waltham, MA, USA) at λ = 414 nm, setting an absorbance value <0.2 as cut-off [46], to distinguish haemolysed from non-haemolysed sera. Haemolysed samples were not included in the cohort.

### 4.3. Total RNA Isolation

Total RNA was isolated from serum using the miRNeasy Serum/Plasma mini kit (Qiagen, Hilden, Germany). Briefly, 800 μL serum was lysed with 5 volumes of Qiazol and processed according to the manufacturer’s instructions. Glycogen (Thermo Fisher Scientific, Waltham, MA, USA), 10 μg, was added to each sample lysate to increase RNA yield. RNA quantification was performed by Nanodrop 1000 (Thermo Fisher Scientific). Total RNA was finally eluted in 30 μL RNase-free water and stored at −80 °C until analysis.

### 4.4. Microarray Analysis

Whole transcriptome analysis was performed on 24 serum samples including 12 T2D patients and 12 unaffected CTRLs. Analysis was performed using the Clariom D Pico Assay (Thermo Fisher Scientific), a technology that analyses the expression of more than 540,000 transcripts, both coding and non-coding, including mRNAs, circRNAs, lncRNAs, and miRNA precursors, as well as other small RNAs. The Pico Assay is optimised for loading a very low input of total RNA, making this platform suitable for serum samples, where low yields of RNA are frequent. The same approach had been previously applied to circulating RNA [28,29]. Specifically, 10 ng total RNA was retrotranscribed in single-stranded cDNA containing the T7 promoter sequence at the 5′ end. We synthesised 3′ double-stranded cDNA by adding an adaptor as a template; the pre-IVT (in vitro transcription) amplification reaction was optimised with 12 cycles of amplification. The double-stranded DNA was used as a template for antisense RNA synthesis and overnight amplification (14 h) by IVT, using T7 RNA polymerase. Approximately 20 μg of purified cRNA were used for sense single-strand cDNA (ss-cDNA) synthesis, followed by RNase H digestion and ss-cDNA magnetic bead purification. Approximately 5.5 μg ss-cDNA was fragmented using uracil DNA-glycosylase (10 U/μL) and apurinic/apyrimidinic endonuclease 1 (1000 U/μL) and then labelled with biotin using terminal deoxynucleotidyl transferase (30 U/μL). From the hybridisation cocktail, 200 μL of the obtained mixture were loaded into a single human Clariom D 49-format array and incubated for 16 h in the Affymetrix GeneChip Hybridization Oven 645 at 45 °C, 60 rpm. Arrays were stained using an Affymetrix GeneChip Fluidics Station 450, according to the specific fluidics protocol (FS450_0001), and scanned with an Affymetrix GeneChip Scanner 3000 7G. Raw intensity CEL files generated by GeneChipTM Command ConsoleTM were imported into the Transcriptome Analysis Console (TAC) 4.0 (Applied Biosystems, Waltham, MA, USA) and CHP files were generated for gene-level analysis. Differentially expressed (DE) transcripts were identified as described in “Statistical analysis”.

### 4.5. Validation in Real-Time PCR

Validation analysis was performed in an independent cohort of 72 individuals (35 T2Ds vs. 37 CTRLs) using Real-Time PCR. Specific PCR primers for the seventeen candidate biomarkers were designed with PrimerBlast (https://www.ncbi.nlm.nih.gov/tools/primer-blast/, accessed on 28 June 2023). Two different transcripts were used as endogenous controls, namely GAPDH (glyceraldehyde-3-phosphate dehydrogenase) and RNU6 (RNA, U6 small nuclear 1); only those transcripts showing differential expression according to both endogenous controls were considered significantly deregulated. All primer sequences are reported in Table 5. PCR reactions were performed on a 7900HT Fast Real-Time PCR System using a Power SYBR^®^ Green RNA-to-Ct^TM^ 1-Step Kit (Thermo Fisher Scientific) [47]. DE transcripts were identified using SDS RQ Manager 2.4 software by applying the 2^−ΔΔCt^ method, and differential expression was expressed as RQ (Relative Quantity); RQ values < 1 were converted into fold change (FC) values by applying the formula −1/RQ.

### 4.6. Evaluation of Diagnostic Accuracy

The diagnostic accuracy of DE transcripts was evaluated by ROC (receiver operating characteristic) curve analysis [28,48]. Specifically, we computed univariable and multivariable ROC curves to investigate the diagnostic accuracy of each DE transcript considered as a single biomarker or combinations of variables including expression data (ΔCts) and/or clinicopathological parameters, aiming to find the best biomarker or signature for T2D diagnosis. All curves were computed using SPSS 23 (IBM, Armonk, New York, NY, USA). For each ROC curve, the area under the curve (AUC), 95% confidence intervals (CIs), and *p*-value were calculated; the Youden method was applied to identify the optimal cut-off, with the associated sensitivity, specificity, accuracy, positive predictive value (PPV), and negative predictive value (NPV). For multivariable curves, binary logistic regression models were built and predicted values were used as input data for ROC curve computation; sensitivity, specificity, accuracy, PPV, and NPV were calculated using confusion matrixes and predicted grouping generated by the regression model. Statistical significance was established at a *p*-value < 0.05 for all ROC curves.

### 4.7. Statistical Analysis

Statistical analysis of profiling data was performed as previously described [28]. DE transcripts were identified with two different statistical approaches. First, the software TAC 4.0.2.15 was used with the following settings: Analysis Type: Expression Gene; Summarisation Method: Gene Level–RMA. Gene-Level *p*-Value < 0.05 ANOVA Method: ebayes. Simultaneously, the software MeV (Multi Experiment Viewer) v4.9.0 was used by applying Significance of Microarrays Analysis (SAM); unpaired tests were performed among ΔCts using a *p*-value based on 100 permutations; imputation engine: K-nearest neighbours (10 neighbours); false discovery rate (FDR) < 0.05. Results from the two approaches were compared, selecting common transcripts for the next validation step. PCR data were analysed using GraphPad Prism 8 (Dotmatics, Boston, MA, USA). First, expression data (ΔCts) were tested for normality of distributions (Anderson–Darling test, D’Agostino and Pearson omnibus normality test, Shapiro–Wilk normality test, and Kolmogorov–Smirnov test) and homogeneity of variance (F test); distributions were considered parametric only if all the applied tests gave not significant *p*-values. According to the results, a parametric (homoscedastic or Welch corrected unpaired *t*-test) or non-parametric (Mann–Whitney test) test was applied to identify statistically significant DE transcripts. The same approach was used to identify statistically significant differences in clinicopathological parameters among the two pathological groups to identify potential confounding factors. To evaluate the potential relationship between transcripts and clinicopathological parameters, correlation analysis was performed by calculating the Pearson or Spearman correlation coefficient, according to the normality of distributions; the *p*-values associated with correlation coefficients were corrected for multiple comparisons (Holm–Sidak method).

## 5. Conclusions

This study identified a new potential biomarker for T2D diagnosis in the transcript TC0800011832 coded by the ASAP1 locus. Although HbA1c represents an optimal biomarker for T2D diagnosis, our data show that the signature of biomarkers including both expression data and clinicopathological parameters may increase diagnostic accuracy, improving the clinical management of patients. The patients enrolled in this study had recently been diagnosed and therefore had not been treated for the disease. As discussed in the Introduction, the need for early diagnosis of T2D is urgent. We propose TC0800011832 (in combination with clinicopathological parameters) as a potential biomarker for early diagnosis of T2D. Prospective studies will determine if this biomarker may be used as a prognostic risk factor for the onset of T2D and applied in the context of 3PM.

## Figures and Tables

**Table 1:** DE transcripts identified by Clariom D profiling. The TAC ID, the gene symbol (if available), the mapping chromosome, and the fold change with the associated *p*-value are shown for each transcript.

TAC ID	Gene Symbol	Chromosome	FC	*p*-Value
TC0100009744	*neelybu*	chr1	−2.42	1.19 × 10^−5^
TC0800008985		chr8	−2.62	3.82 × 10^−5^
TC0100013004	*RP4-680D5.2*	chr1	1.62	4.25 × 10^−5^
TC1700010637	*KRT26*	chr17	1.87	6.92 × 10^−5^
TC1600006522	*feyzu*	chr16	−1.82	8.84 × 10^−5^
TC1800006511		chr18	−1.54	9.72 × 10^−5^
TC0900008473		chr9	−2	0.0001
TC1600008350		chr16	−1.76	0.0001
TC0800011832		chr8	−1.59	0.0002
TC0900007337		chr9	−1.92	0.0002
TC0X00007972		chrX	−1.95	0.0002
TC0500010990	*CTC-498J12.1*	chr5	−4.46	0.0003
TC1000007406		chr10	−4.06	0.0003
TC1700011697	*CD300LB*	chr17	−1.42	0.0003
TC2100008031		chr21	−1.43	0.0003
TC1900008595		chr19	−1.25	0.0017

**Table 2:** Results of Real-Time PCR validation. For each transcript, identified with the TAC ID and the gene symbol (if available), the FC obtained in microarray analysis is shown, together with the median fold change from validation and the associated *p*-value (between brackets) for each endogenous control (GAPDH and RNU6). Significant values are highlighted in bold.

TAC ID	Gene Symbol	FC Profiling	GAPDH	RNU6
TC0100013004	*RP4-680D5.2*	**1.62**	1.02 (0.28)	−1.11 (0.94)
TC0500010990	*CTC-498J12.1*	**−4.46**	1.1 (0.65)	−1.04 (0.78)
TC0800008985		**−2.62**	1.02 (0.78)	−1.10 (0.46)
TC0800011832		**−1.59**	**−1.49 (0.034)**	**−1.54 (0.006)**
TC0900007337		**−1.92**	1.08 (0.46)	−1.05 (0.72)
TC0900008473		**−2**	−1.33 (0.32)	−1.21 (0.13)
TC1000007406		**−4.06**	−1.07 (0.35)	−1.21 (0.62)
TC1600006522	*feyzu*	**−1.82**	1.12 (0.21)	−1.01 (0.65)
TC1600008350		**−1.72**	−1.08 (0.58)	−1.22 (0.14)
TC1700011697	*CD300LB*	**−1.42**	1 (0.65)	−1.14 (0.61)
TC1800006511		**−1.54**	−1.07 (0.76)	−1.21 (0.55)
TC1900008595		**−1.25**	1.00 (0.25)	−1.18 (0.66)
TC2100008031		**−1.43**	−1.07 (0.39)	−1.26 (0.13)

**Table 3:** Results of ROC curve analysis. Univariable and multivariable ROC curve data are reported: the *p*-value, the AUC, its standard error (Std error), and 95% CIs are shown for each computed curve; sensitivity, specificity, accuracy, PPV, and NPV are also shown. For univariable ROC curves, the ΔCt cut-off discriminating T2D and CTRL is shown.

Variables	TC0800011832		Clinical Data	TC0800011832 + Clinical Data		All Transcripts		All Transcripts + Clinical Data	
**Endogenous control**	GAPDH	RNU6	/	GAPDH	RNU6	GAPDH	RNU6	GAPDH	RNU6
**AUC**	0.649	0.692	0.973	0.972	0.975	0.71	0.723	1	1
**Std error**	0.067	0.065	0.02	0.021	0.02	0.062	0.06	0	0
***p*-value**	0.034	0.006	7.34 × 10^−9^	1.34 × 10^−8^	6.59 × 10^−9^	0.002	0.001	1.75 × 10^−9^	9.95 × 10^−10^
**95% CI min**	0.517	0.565	0.933	0.93	0.936	0.59	0.605	1	1
**95% CI max**	0.781	0.819	1	1	1	0.831	0.84	1	1
**Cut-off**	4.588	2.116	/	/	/	/	/	/	/
**Accuracy**	0.68	0.7	0.94	0.94	0.94	0.66	0.65	1	1
**Sensitivity**	47.06	73.53	0.94	0.94	0.94	0.60	0.6	1	1
**Specificity**	88.57	66.67	0.95	0.95	0.95	0.72	0.7	1	1
**PPV**	0.8	0.68	0.97	0.97	0.97	0.68	0.66	1	1
**NPV**	0.63	0.73	0.91	0.9	0.91	0.65	0.65	1	1

**Table 4:** Clinicopathological parameters of the entire cohort of 96 individuals divided into pathological groups (T2D and CTRL). Data are presented as average ± standard deviation. BMI: body mass index; AST: aspartate transaminase; ALT: alanine transaminase; PLT: platelet count; HDL: high-density lipoprotein; LDL: low-density lipoprotein; HbA1c: haemoglobin A1c; uACR: urine albumin-creatinine ratio; WBC: white blood cells; HCT: haematocrit test.

Parameter	T2D	CTRL
Age	59.3 ± 8.3	55.8 ± 9.4
Sex (M:F)	31:16	27:22
BMI	28.1 ± 5.5	27.6 ± 5.1
Height (m)	1.66 ± 0.09	1.68 ± 0.09
Weight (kg)	77.5 ± 16.8	77.7 ± 17.9
Waist (cm)	99.3 ± 14.3	89 ± 21.7
AST (IU/L)	27.2 ± 11.5	28.4 ± 10.4
ALT (IU/L)	34 ± 25.2	30.4 ± 17.9
PLT/μL	227,131.9 ± 73,568	234,042.6 ± 52,032.4
Total cholesterol (mg/dL)	179.2 ± 34.5	197.5 ± 29.3
HDL (mg/dL)	52.8 ± 11.1	55.3 ± 14.1
LDL (mg/dL)	100.7 ± 29.9	116.3 ± 34.2
Triglycerides (mg/dL)	128.3 ± 68.8	123 ± 88
Uric acid (mg/dL)	5 ± 1.2	5.1 ± 1.8
HbA1c (%)	7.2 ± 1.1	5.6 ± 0.3
Creatinine (mg/dL)	0.7 ± 0.1	0.8 ± 0.1
Urinary albumin (mg/mL)	16.1 ± 18	15 ± 13.1
uACR	15.4 ± 18.3	17.1 ± 14.9
WBC/μL	7330 ± 2323.4	6522.9 ± 1931.5
HCT (%)	43.2 ± 6	43.5 ± 3.5

**Table 5:** PCR primers used for validation of profiling results.

Transcript	Forward Primer	Reverse Primer
CD300LB	CAACAGCAAGCTCACCTACCA	GTAGGGGCAGGAGAAAGAACC
CTC-498J12.1	GCACTATTGATTCCTGCCCCA	CCTGGCCCCAACAAACTACA
feyzu	CCCGAGCTGGCTGAGACATA	TCATCAACACGCACCTCTGC
GAPDH	TGCACCACCAACTGCTTAGC	GGCATGGACTGTGGTCATGAG
KRT26	GCCAGAAATGAGCTGACCGAAT	TCAGTCTCAGCCAAGGAGCATT
neelybu	CAACCTAGTCCCGTTGAACACA	ACACCAGAGGCTGGGTATTGA
RNU6	CTCGCTTCGGCAGCACA	AACGCTTCACGAATTTGCGT
RP4-680D5.2	GCAAGAAAGTGGGGGCTGAG	CTGCCCGGTAATGCTTCCTG
TC0800008985	CTGCATGGGGGCAGTAAGTG	CTGTTCCACCCCTCCAGACT
TC0800011832	TCCACACTGCTGAAAAATCTGGT	CCAAGGATTGAGGGGGAAGGA
TC0900007337	TGTAGCATCACCTGGGAGGG	GGTGTGGTGTTTTGTGCAGC
TC0900008473	AAGCCTCCTACCCTGCCAAT	TCCAGGTGAGGTGACTTGCT
TC0X00007972	TGTCCCCACATCACTCACTGG	GGCAGGTCACAATGGGGTATTC
TC1000007406	ACGAATAGCCCCATCAGGGA	AGAGCACATTGCACGCAGG
TC1100010184	AAAGGTGCCAAAGAAAAGGCAG	TGAAAGCCAGAAAATAGCCACCT
TC1600008350	GTAAGGGCTTCAGGCTGCTTC	GCAAACCCCAACTCCGCTT
TC1800006511	CACCCACATTCCATACAGCCTT	GCAGGGCACCATGAGAAGTAA
TC1900008595	GCAGAAAGGCTTGTGGCTTCA	TCTTCACACTGCTCTCCCTTACG
TC2100008031	TCCCTAACGCACCTCTTGCT	TGAGGAAACTGAGGGCACCA

## Data Availability

All data reported in this paper are available from the corresponding author.

## Supplementary Materials

The supporting information can be downloaded at: https://www.mdpi.com/article/10.3390/ijms241713485/s1.
